# Sensing and Induced Transparency with a Synthetic Anti-PT Symmetric Optical Resonator

**DOI:** 10.1021/acsomega.0c05673

**Published:** 2021-02-13

**Authors:** Haoye Qin, Yiheng Yin, Ming Ding

**Affiliations:** † School of Instrumentation and Opto-Electronics Engineering, Beihang University, Beijing 100191, China; ‡ School of Microelectronics, Beihang University, Beijing 100191, China; § Research Institute of Frontier Science, Beihang University, Beijing 100191, China

## Abstract

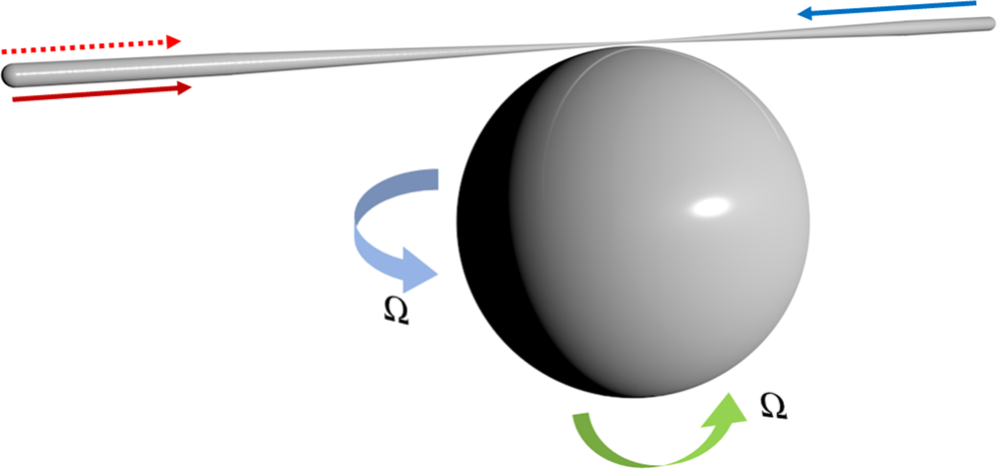

Synthetic dimensions and anti-parity-time (anti-PT) symmetry have been recently proposed and experimentally demonstrated in a single optical resonator. Here, we present the effect of the rotation-induced frequency shift in a synthetic anti-PT symmetric resonator, which enables the realization of a directional rotation sensor with improved sensitivity at an exceptional point (EP) and transparency assisted optical nonreciprocity (TAON) in the symmetry-broken region. The orthogonal rotation of this system results in the direction-independent frequency shift and maintenance of the EP condition even with rotation. Tunable transparency at the EP can thus be fulfilled. Hopefully, the proposed mechanisms will contribute to the development of high-precision rotation sensors and all-optical isolators and make the study of the synthetic anti-PT symmetric EP with rotation possible.

## 1 Introduction

Non-Hermitian optics has attracted great interest for its rich physics and applications.^1,2^ Optical whispering gallery mode (WGM) resonators provide a ready and controllable platform to realize parity-time (PT) and anti-PT symmetry.^3^ Such experimental systems commonly involve two coupled resonators with a balanced gain–loss rate. At an exceptional point (EP), the eigenvalues of Hamiltonian coincide in their real and imaginary parts, which can be achieved via tuning gain–loss contrast^4,5^ or manipulating the backscattering in one resonator, e.g., the introduction of nanoparticles within the evanescent field of WGM and tuning their positions.^4,6^ These lead to distinctive phenomena like enhanced sensing capability,^1,7,8^ nonreciprocal light transmission,^5,9,10^ nonadiabatic transitions of EP encircling,^11,12^ chiral optical states,^4^ and transparency at the EP.^4,6^ Notably, EP gyroscopes have been put forward for rotation sensing with enhanced Sagnac sensitivity.^13−15^ Extension of the original parity operator has been proposed to encompass any linear operator including rotation and inversion.^16^ Recently, Wan and co-workers^17^ presented the theoretical and experimental demonstration of the synthetic anti-PT symmetry and EP in a single optical resonator using stimulated Brillouin scattering (SBS),^18^ providing an insight into the synthetic spectral dimensions.^19^ Besides, the stationary WGM resonators have been pushed into movements like spinning and parallelly vibrating for novel investigations.^20−23^ Employing a spinning resonator can provide enhanced nanoparticle sensitivity,^20^ tunable optomechanically induced transparency,^21^ and optical nonreciprocity.^22^ Several pioneering arts have been put forward in nonreciprocal optics, for instance, nonreciprocal phonon lasers^24^ and photon blockades.^25,26^ A famous experiment has demonstrated the nonreciprocal light propagation in PT symmetric resonators,^27^ with the fundamental theory explain by ref (28). However, the application potential of both spinning resonators and synthetic anti-PT symmetry is still merely revealed and underdeveloped. There exist distinctive features like sensing capability and optical nonreciprocity indicated by the EP or rotation-induced irreversible refraction waiting to be explored.

Therefore, in this work, we theoretically investigate the rotation in a synthetic anti-PT symmetric optical resonator mentioned in ref (14). This resonator can work as a rotation sensor with enhanced sensitivity and capability of determining the rotation direction at the EP in synthetic anti-PT symmetry at a relatively slow velocity. Also, due to the direction-dependent frequency shift from in-plane rotation, transparency and absorption-assisted optical nonreciprocity (AAON) in the rotation-induced symmetry-broken region have been proposed and analyzed. Finally, the illustration of the synthetic anti-PT symmetric resonator rotating orthogonally is provided for the new direction-independent frequency shift and maintenance of the EP condition at the rotation state, accompanied by the tunable transparency at the EP. The proposed mechanisms are promising for achieving high-precision wideband directional rotation sensing and more efficient optical isolator devices. In addition, orthogonal rotation breaks the nonreciprocity and enables the study of synthetic anti-PT symmetry and its EP at the moving condition.

## 2 Results and Discussion

**Figure 1:**
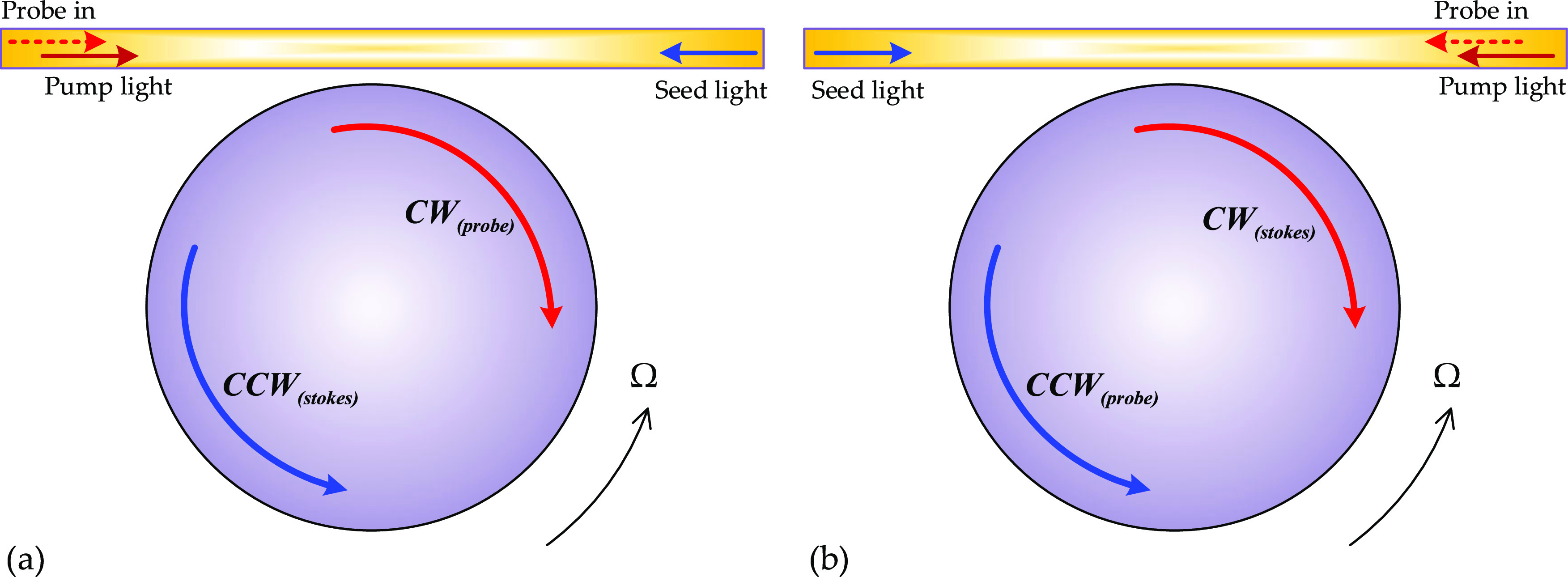
Schematic diagram of a rotating synthetic anti-PT-symmetric optical resonator with the excitation method. Due to the SBS process, there exist two counterpropagating modes, CW and CCW modes. The resonator is subject to mechanical rotation at a velocity of Ω. The cases of the forward probe (a) and the backward probe (b).

As shown in Figure 1, our consideration is based on an optical resonator loaded with the mode excitation and coupling mechanism of Brillouin scattering-induced transparency (BSIT),^17^ i.e., the counterpropagation of pump light and seed light is inserted via a tapered fiber, with weak probe light (at a frequency of ω_p_) sweeping its frequency. Specifically, Figure 1a presents the case of the forward probing scheme and Figure 1b shows the backward one, which have been employed to demonstrate the system’s nonreciprocity. Above a certain power threshold and through the electrostrictive-induced SBS process, the probe light can stimulate the generation of Stokes photons (at a frequency of ω_s_, confined in the CCW mode) and coherent acoustic phonons with a frequency of ω_a_ = ω_p_ – ω_s_. The formed acoustic wave^17,29,30^ then behaves as a middleman for tailoring a nonlinear coupling between the probe field (as the CW mode) and the Stokes field. Besides, rotation (angular velocity Ω) is introduced to the resonator either via manually spinning it or via operating as a gyroscope, which leads to an induced Sagnac-Fizeau frequency shift^21,22^ expressed by
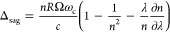
1where *n* is the refractive index of silica, *c* is the speed of light in vacuum, and *R* is the radius of the resonator. The dispersion term  occupies only 1% of the former part.^20^ ω_c_ represents the resonance frequency for a stationary resonator. It is worth noting that the value of Ω has a sign determined by the CW or CCW rotation. For simplicity, the frequency shift is regarded as the same for the probe and Stokes modes under the condition of ω_p_ ≫ ω_a_.

We define the detuning Δ_p,s_ = ω_p,s_ – ω_p0,s0_, which is highly tunable via the probe and seed lasers, with ω_p0,s0_ representing the neighboring WGM frequency of the probe and seed light. The coupling loss induced by the tapered fiber is defined as γ_ex_. With *A*_p,s_ denoting the modal amplitude of the pump and seed light, the coupled mode equation^31,32^ can then be written as
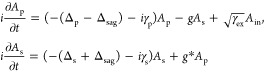
2where γ_p,s_ is the optical loss for the pump and seed light and *g* is the coupling strength, leading to the Hamiltonian of the system.
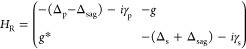
3

To transform it into the synthetic anti-PT symmetry, we rewrite *A*_p_ = *a*_p_ exp(*i*(Δ̅ + *i*γ_p_)*t*) and *A*_s_ = *a*_s_ exp(*i*(Δ̅ = *i*γ_s_)*t*) in the coupling theory;^17^ thus
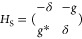
4where
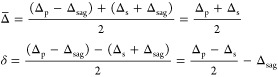
5

*H*_S_ demonstrates the synthetic anti-PT symmetry verified by the anticommutation {*H*_S_, *PT*} = 0 and the commutation [*H*_S_, *P*_S_*T*] = 0, with the synthetic parity operator^17^ defined by
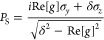
6where *σ_y,z_* are two Pauli matrices for *y* and *z*.

Eigenvalues of *H*_S_ are
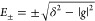
7

At the stationary state, by tuning the system to the exceptional point (EP) condition, i.e., |Δ_p_ – Δ_s_| = 2|*g*|, once there is external rotation, this system will support two supermodes around both probe and Stokes resonances. Their beat frequency is Δω_b_ = |Re[Δ*E*] = |Re[*E*_+_ – *E*_–_]| and for operation of gyroscope (|Δ_sag_| ≪ |Δ_p_ – Δ_s_|)
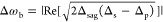
8

**Figure 2:**
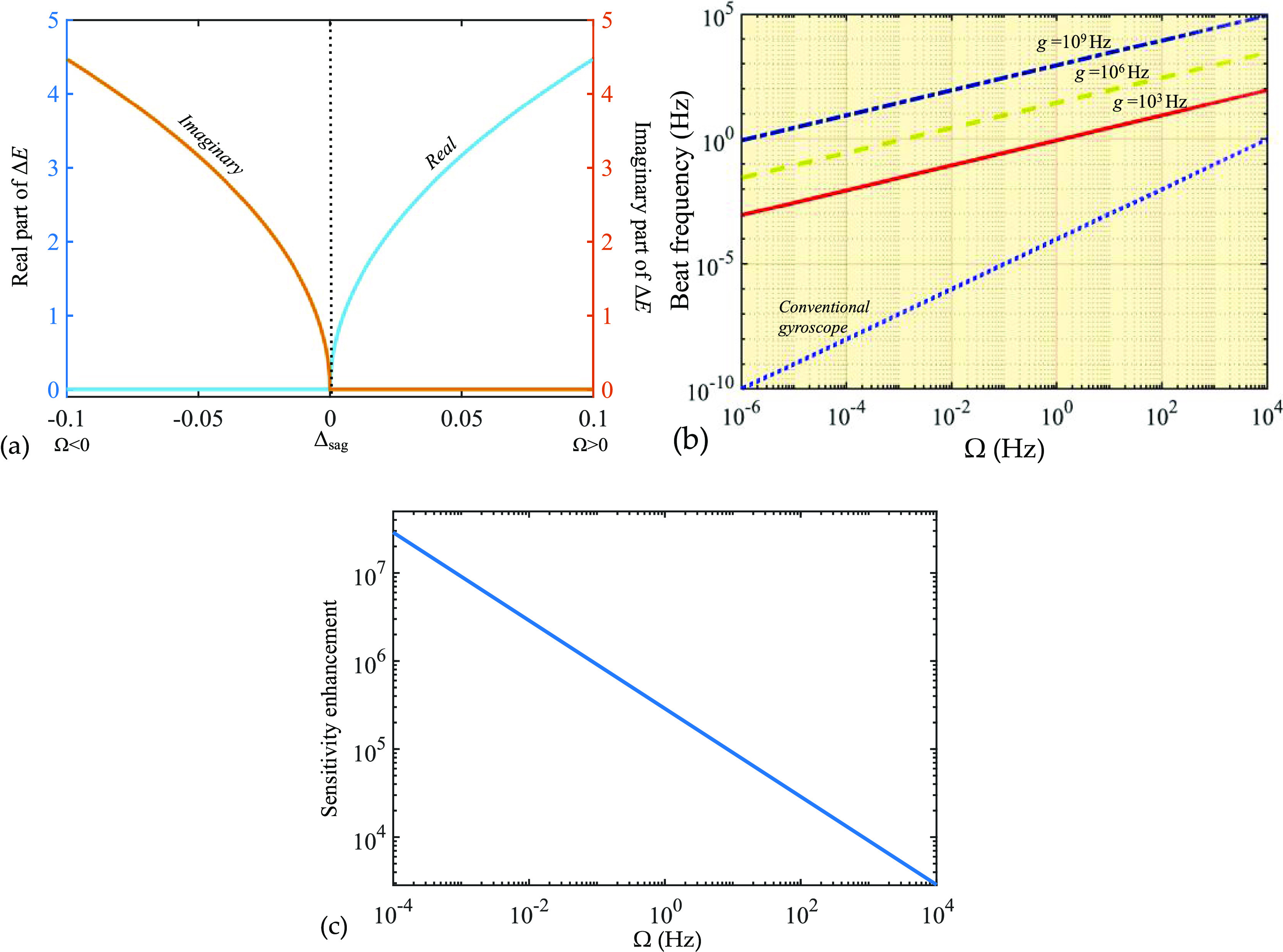
System’s response due to spinning perturbation at the EP condition of |Δ_p_ – Δ_s_| = 2|*g*|. (a) The real (pink line) and imaginary parts (red line) of Δ*E* versus Δ_sag_. (b) Beat frequency versus rotation velocity for the synthetic anti-PT symmetric resonator (upper lines, with three different coupling rates *g* = 10^3^, 10^6^, and 10^9^ Hz) and a conventional resonator gyroscope (lower line). (c) Sensitivity enhancement of the synthetic anti-PT symmetric resonator (*g* = 10^6^ Hz) with increasing rotation velocity compared with the conventional one.

The rotation sensing of this synthetic anti-PT symmetric resonator is direction-dependent and can be employed to detect the direction of rotation. The mechanism of direction dependence is explained as follows. Assuming Δ_s_ > Δ_p_, Figure 2a plots the real and imaginary parts of Δ*E* versus different Δ_sag_. When the rotation velocity is negative (e.g., CW rotation), the imaginary part is greater than zero and the real part is equal to zero; when the rotation velocity is positive (e.g., CCW rotation), the real part is greater than zero and the imaginary part is equal to zero. Therefore, in the spectral response, CCW rotation will cause mode splitting, and reverse rotation will cause no splitting effect. For detecting the reverse rotation, one just needs to adjust the value of Δ_p_ to be greater than Δ_s_.

To be more specific, experimental feasible parameters are chosen for the optical resonator as: *R* = 250 μm, *n* = 1.44, and ω_c_ = 194 THz. In Figure 2b, the beat frequency as a function of the rotation velocity is demonstrated. The lower line refers to a conventional resonator gyroscope and the upper three lines indicate the case of the synthetic anti-PT resonator with different coupling rates *g* = 10^3^, 10^6^, and 10^9^ Hz. There is a great enhancement in the synthetic anti-PT resonator in detecting the slow rotation due to the square-root feature of Δω_b_. At a typical value of *g* = 10^6^ Hz, the sensitivity enhancement presents a decreasing trend with a larger rotation velocity (Figure 2c), limiting the advantage of this kind of rotation sensing only to a slow angular velocity. Since this is a second-order EP, the sensitivity enhancement would be comparable with other second-order systems but lower than higher-order ones.^8^ However, our advantages exhibit in the tunable sensing direction and capability via the term Δ_s_ – Δ_p_ and also the basis in the synthetic anti-PT domain.

Manually spinning the resonator will lead the EP achieved at the stationary condition to either the synthetic anti-PT symmetric (PT broken) region or the PT symmetric (synthetic anti-PT broken) region. In these symmetry-broken regions, the transparency assisted optical nonreciprocity (TAON) is proposed for light isolation.

Considering the coupled mode equations for the two conditions shown in Figure 1 (the first group of equations for Figure 1a and the second for Figure 1b)
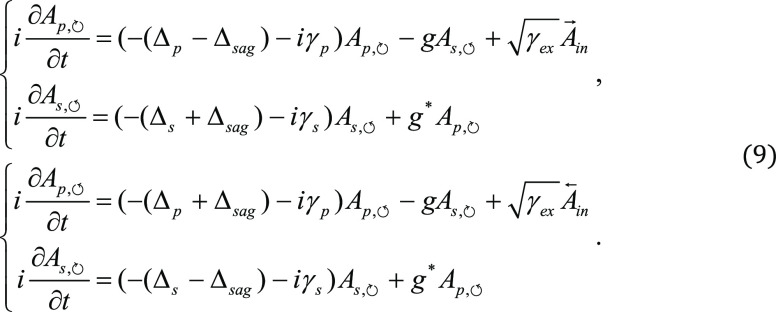
9 where the denotation 
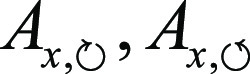
 represent CW and CCW of the *x* (probe and Stokes) mode and  represent forward and backward *y* (input; output) fields, respectively. Using the standard input–output relation, the corresponding transmission of probe light is given by
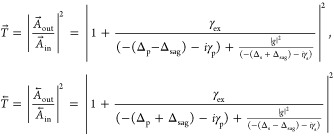
10where  are the forward and backward probe transmission rates, respectively.

**Figure 3:**
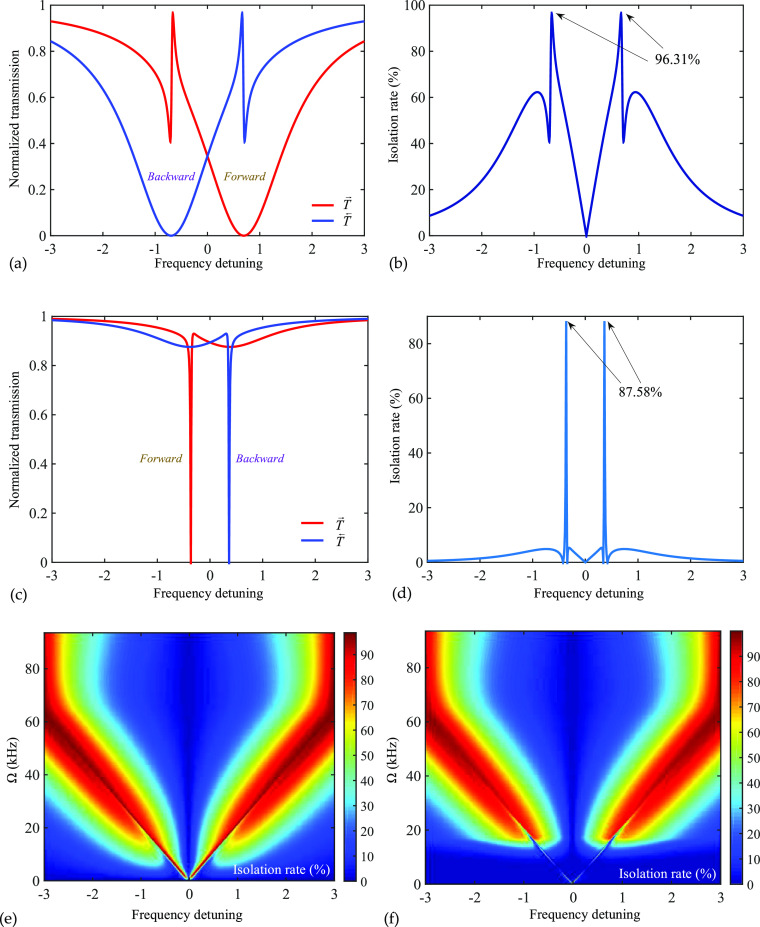
Spectrum of transparency (a) and absorption (c) assisted optical nonreciprocity. Here, the red line indicates the transmission spectrum for the forward probe  and the blue line indicates the transmission spectrum for the backward probe . (b) The calculated isolation rate of (a) with a maximum of 96.31 %. (d) The calculated isolation rate of (c) with a maximum of 87.58 %. The frequency detuning is defined as probe cavity detuning Δ_p_ and normalized to γ_p_. The isolation rate as a function of frequency detuning and rotation velocity for TAON (c) and AAON (d). Δ_p_ is assumed to be equal to Δ_s_. Simulation parameters: (*a*, *b*, *e*) γ_ex_ = 0.01γ_p_, γ_s_ = 0.01γ_p_, *g* = 0.2γ_p_, γ_p_ = 6.25 × 10^5^ Hz, and Ω = 6.9 kHz; (c, d, f) γ_ex_ = 0.01γ_p_, γ_s_ = −0.01γ_p_, *g* = 0.16γ_p_, γ_p_ = 6.25 × 10^5^ Hz, and Ω = 3.6 kHz.

Figure 3a shows the transmission spectrum for the forward probe  and the backward probe  at a rotation velocity of 6.9 kHz. The transparency peak of  is aligned in accordance with the resonance dip of , resulting in the maximum passed forward light and blocked backward light. We call this effect transparency assisted optical nonreciprocity (TAON) and it occurs symmetrically at about zero detuning. In Figure 3b, the related isolation rate is numerically calculated versus frequency detuning via the definition of pass transmission minus block transmission. The peak of the isolation rate is positioned at the transparency, reaching over 96%. Compared with previous works achieving nonreciprocity using rotation and only optical resonance dip, the TAON has an advantage over a smaller required rotation velocity under the same condition. Also, this mechanism is more suitable for a low-quality factor resonator due to the assistance of sharp transparency.

Similarly, absorption-assisted optical nonreciprocity (AAON) can be realized at a rotation velocity of 3.6 kHz, as shown in Figure 3c for the forward–backward transmission spectrum and in Figure 3d for the corresponding isolation rate. The sharp absorption of the one input direction is positioned at the resonance dip of the other input direction. Intuitively, the isolation of AAON is not so perfect as that of TAON. Symmetry at about zero detuning persists considering that the rotation-induced shift is direction-dependent for the CW and CCW modes. The isolation rate as a function of frequency detuning and rotation velocity is provided in Figure 3e,f for TAON and AAON, respectively. The linear relation between the rotation velocity and the frequency shift is clearly shown in the V-shaped bifurcation, around which the isolation rate is approximately 100%. This pattern can be attributed to the linear relation between the rotation velocity and the Sagnac frequency shift, as indicated in eq 1 and in the experimental results of ref (22).

TAON and AAON occurring in the symmetry-broken region may open new avenues for all-optical isolators, reducing the rotation velocity for spinning-resonator-based optical nonreciprocity. Their isolation capability is better than that of a Hermitian spinning resonator^22^ and closed to ref (10) but with more tunable features. Understood as a combination of the Sagnac effect and induced transparency, this nonreciprocity can be readily tunable via parameters to fit different applications. To improve the operation bandwidth for practical isolators, *g* and γ_s_/γ_p_ could be increased since they reduce the sharpness of transparency lineshape. Also, as shown in Figure 3e,f, a smaller Ω (<15 kHz) features a rather limited isolation bandwidth (indicated by the area of red color) and improved Ω causes the bandwidth broadening, while an extremely low bandwidth may still be useful to work as an isolation switch, which is achievable through reversely tuning the above parameters.

As for the abovementioned rotation, confined within the reference plane, the Sagnac frequency shift is introduced anisotropically for counterpropagating modes. The drawbacks of this rotation include the rotation-induced broken EP condition and decreased response for a large spinning velocity (Figure 2c). It is novel and important to consider another type of rotation, rotating orthogonally, of the synthetic anti-PT resonator, to manage to achieve spinning EP and the enhanced response of a larger velocity.

**Figure 4:**
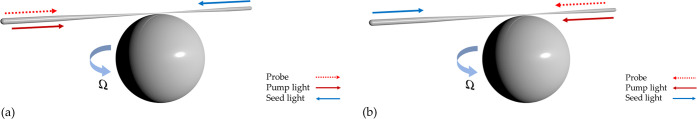
Schematic illustration of an orthogonally rotating synthetic anti-PT-symmetric optical resonator. The cases of the leftward probe (a) and the rightward probe (b). The inset shows the denotation of injected optical fields.

**Figure 5:**
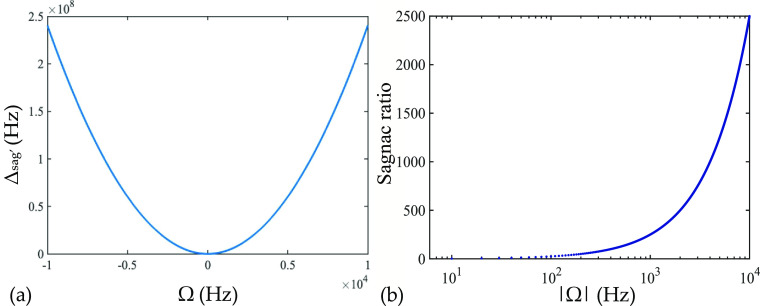
(a) New frequency shift versus rotation velocity for orthogonal rotation. (b) Sagnac ratio (SR) as a function of the absolute value of the rotation velocity.

With Figure 4a showing the schematic diagram of an optical resonator rotating orthogonally, to obtain the rotation-induced frequency shift, we employ the relativistic speed formula for the light traveling along the circumference. The resultant new frequency shift is
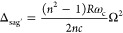
11where expected Ω^2^ occurs indicating a direction-independent value in contrast with eq 1. Figure 5a displays the relation between the rotation velocity and the value of the frequency shift.

To compare the frequency shift induced by two types of rotation, we define a Sagnac ratio (SR) given as
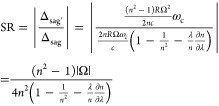
12

Figure 5b presents the calculated value of SR versus the angular velocity of rotation, where we observe a dramatical enhancement of SR with increasing angular velocity. A larger SR represents that there will be more rotation-induced frequency shift in the orthogonally rotating resonator than in the resonator rotating confinedly in the plane, that is, higher rotation sensitivity for the former. Therefore, based on the synthetic anti-PT EP at a slow velocity and a considerable SR at a fast velocity, a gyroscope incorporating both in-plane and orthogonal rotations can be conceived for the improved sensing capability and a wider detection range.

In the following, the effect of orthogonal rotation on the synthetic anti-PT symmetry and optical nonreciprocity is evaluated. Due to the direction independence of this new shift, both CW and CCW modes will have a detuning with the same sign, i.e., ω_s0,p0_ → ω_s0,p0_ – Δ_sag′_ and the corresponding parameters in *H*_S_ and EP conditions are rewritten by
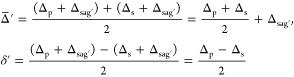
13

A close inspection of δ′ and the EP condition produces a conclusion that a stationary resonator at the EP will still hold the EP when rotating orthogonally. It is important to note that here the EP refers to both synthetic PT and anti-PT symmetries.

**Figure 6:**
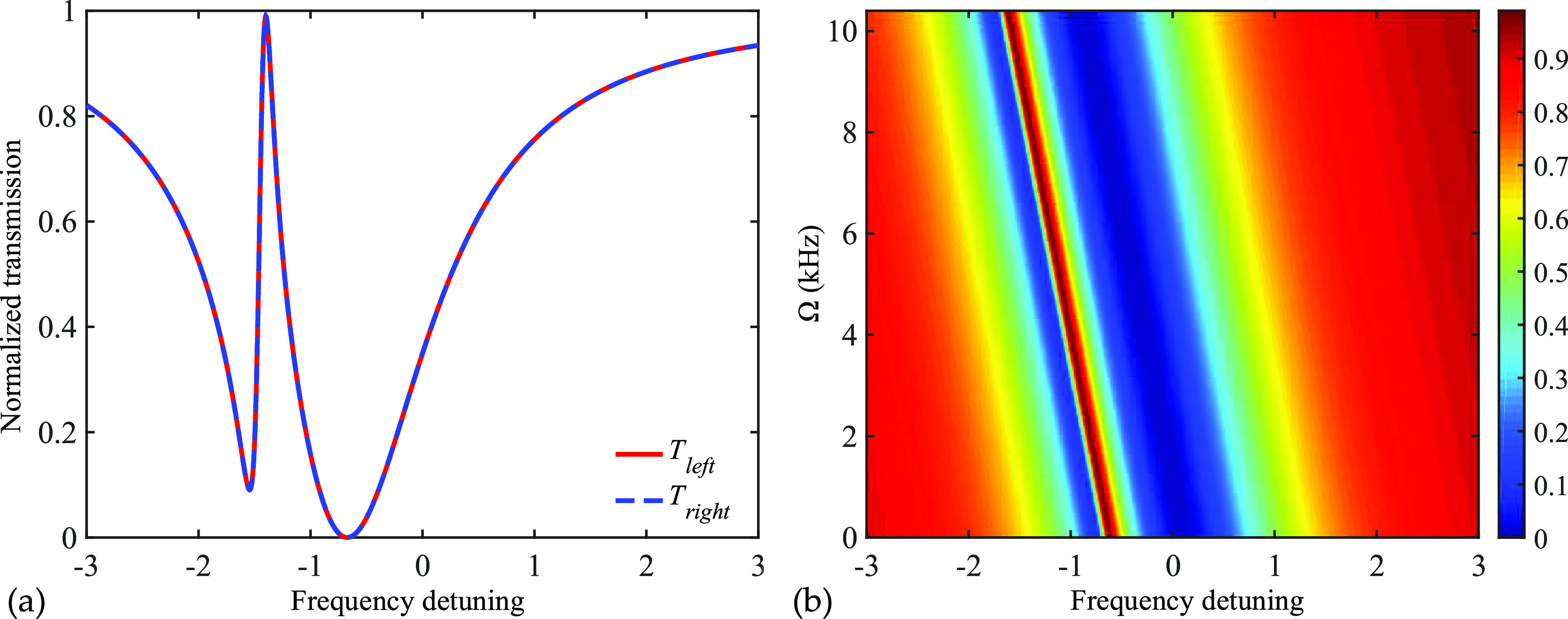
(a) Transmission spectrum for the leftward (the red line) and rightward (the blue dashed line) probes. Transparency occurs at the stationary EP condition. (b) Transmission as a function of frequency detuning and rotation velocity, which shows a signature of tunable transparency at the EP via the rotation velocity. The frequency detuning is normalized to γ_p_. Simulation parameters: γ_ex_ = 0.01γ_p_, γ_s_ = 0.01γ_p_, *g* = 0.3γ_p_, γ_p_ = 6.25 × 10^5^ Hz, and Δ_p_ = Δ_s_ + 2*g*.

The scheme of loading the leftward and rightward probes is given in Figure 4. Using the coupled mode equations for both cases, the analytic solution of probe transmission is
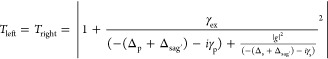
14which is the same for two counterpropagating inputs. Figure 6a displays the coalesced transmission spectrum at Δ_sag′_ = 0.8 (Ω = 4.16 kHz), with *T*_left_ and *T*_right_ experiencing the same frequency shift, and thus there is no nonreciprocal propagation. The EP condition is satisfied by choosing the relation of parameters as Δ_p_ = Δ_s_ + 2|*g*|. Notably, this is also a kind of transparency at EP. Since the system remains at the EP regardless of being stationary or rotating, angular velocity can be employed for tunable transparency at the EP. Figure 6b shows the results of transparency at the EP with different values of Ω. Herein, this synthetic anti-PT resonator may be employed as a platform for studying the maintained EP at the rotation state. Therefore, both rotation-induced nonreciprocal and reciprocal propagations can be realized in the system by controlling the rotation state of resonators, which has not been demonstrated anywhere else.

In the end, we discuss the feasibility of experimentally implementing such a synthetic anti-PT resonator for enhanced sensing and nonreciprocal light propagation. The first spinning optical resonator has been successfully demonstrated using a commercial motor, and the coupling between the taper and the resonator maintains enough stability by forming the so-called flying coupler, with the assistance of the aerodynamic process.^22^ The introduction of the Sagnac frequency shift is proved in the nonreciprocal transmission feature and fitted as theoretically predicted. Thanks to the pretty mature fabrication procedures of both spherical and toroidal cavities, the impact of shape imperfection and surface roughness can be greatly suppressed and thus is negligible. In addition, detailed analysis has been established to describe this spinning process,^10,20,22^ which guarantees the experimental feasibility.

## 3 Conclusions

In summary, we have demonstrated the spinning effects in a synthetic anti-PT symmetric optical resonator, with the rotation velocity ranging from relatively small to large. The exceptional point of the synthetic anti-PT symmetry contributes to enhanced rotation sensing capability and enables the determination of the rotation direction. To advance, pushing the system into the symmetry-broken region via tuning the velocity results in optical nonreciprocity assisted by transparency and absorption, which is promising for developing all-optical isolators and circulators. Also, the orthogonal rotation of the resonator is analyzed for its amount of frequency shift, direction independence, and improved rotation sensitivity at a fast velocity. Correspondingly, nonreciprocity due to irreversible refraction disappears and tunable transparency at the EP can be realized. Hopefully, the proposed mechanism will help in designing high-sensitivity wide-range rotation sensors and investigating the synthetic anti-PT symmetry.

## Appendix A

Under the assumptions that light travels along the circumference of the microsphere and the surface velocity remains perpendicular to the light, the velocity in the laboratory frame (*v*) can be given as

15

where *u*(θ) = *R*·Ω·sin θ is the surface speed at angle θ of the great circle, *n* is the refractive index of silica, *R* is the radius of the microsphere, and *c* represents the light speed in vacuum.

The total optical path length can be obtained as

16

Therefore, the new frequency shift for orthogonal rotation is

17
